# A Pilot Study of Immune Activation and Rifampin Absorption in HIV-Infected Patients without Tuberculosis Infection: A Short Report

**DOI:** 10.1155/2017/2140974

**Published:** 2017-12-21

**Authors:** Christopher Vinnard, Isabel Manley, Brittney Scott, Mariana Bernui, Joella Adams, Sheryl Varghese, Isaac Zentner, Michele A. Kutzler

**Affiliations:** ^1^Public Health Research Institute, Rutgers, The State University of New Jersey, Newark, NJ, USA; ^2^Division of Infectious Diseases & HIV Medicine, Department of Medicine, Drexel University College of Medicine, Philadelphia, PA, USA; ^3^Brown University School of Public Health, Providence, RI, USA

## Abstract

**Background:**

Rifampin malabsorption is frequently observed in tuberculosis patients coinfected with human immunodeficiency virus (HIV) but cannot be predicted by patient factors such as CD4+ T cell count or HIV viral load.

**Methods:**

We sought to describe the relationship between HIV-associated immune activation, measures of gut absorptive capacity and permeability, and rifampin pharmacokinetic parameters in a pilot study of 6 HIV-infected, tuberculosis-uninfected patients who were naïve to antiretroviral therapy.

**Results:**

The median rifampin area under the concentration-versus-time curve during the 8-hour observation period was 42.8 mg·hr/L (range: 21.2 to 57.6), with a median peak concentration of 10.1 mg/L (range: 5.3 to 12.5). We observed delayed rifampin absorption, with a time to maximum concentration greater than 2 hours, in 2 of 6 participants. There was a trend towards increased plasma concentrations of sCD14, a marker of monocyte activation in response to bacterial translocation, among participants with delayed rifampin absorption compared to participants with rapid absorption (*p* = 0.06).

**Conclusions:**

Delayed rifampin absorption may be associated with elevated markers of bacterial translocation among HIV-infected individuals naïve to antiretroviral therapy. This trial is registered with NCT01845298.

## 1. Background

Standardized antituberculosis drug regimens that include rifampin, isoniazid, pyrazinamide, and ethambutol are the cornerstone of the global public health response to the tuberculosis epidemic [1]. There is wide variability in the absorption and metabolism of the antituberculosis drugs, and low antituberculosis drug concentrations in blood are associated with inferior tuberculosis treatment outcomes, including treatment failure and relapse [2–5]. Pharmacokinetic variability has been identified as a key mediator of the rate of sterilizing effect and the emergence of new drug resistance mutations during tuberculosis therapy [6, 7]. Among the first-line antituberculosis drugs, rifampin malabsorption is frequently observed among tuberculosis patients coinfected with HIV but cannot be predicted by factors such as CD4+ T cell count, viral load, or the presence of diarrhea [8, 9]. In clinical studies of tuberculosis patients without HIV coinfection, decreased intestinal absorptive capacity and increased intestinal permeability, which may be clinically inapparent, were related to malabsorption of antituberculosis drugs, including rifampin [10–12].

The gut-associated lymphoid tissue is the site of early and dramatic lymphocyte depletion in HIV-infected patients, with near complete loss of intestinal CCR5+ CD4+ T cells within the first few weeks of infection [13]. Lymphocyte depletion is accompanied by the loss of intestinal epithelial barrier integrity, and recent attention has focused on potential role of translocation of lipopolysaccharide and other bacterial products across the damaged intestinal barrier [14]. In support of this model, systemic immune activation markers have been shown to predict HIV progression better than CD4+ T cell count or HIV viral load [15]. Among individuals with chronic HIV infection, levels of soluble CD14 (sCD14), a marker of monocyte response to lipopolysaccharide, were an independent predictor of mortality [16].

The impact of HIV disease on the patient's capacity to absorb specific drugs and nutrients merits further investigation, with a particular need to understand variability in rifampin absorption in relation to immunologic parameters [17]. We sought to describe the relationship between HIV-associated immune activation, measures of intestinal absorptive capacity and permeability, and rifampin absorption among HIV-infected individuals naïve to antiretroviral therapy.

## 2. Methods

We performed a nonrandomized, open-label, pilot study of rifampin pharmacokinetics in 6 HIV-infected adults (aged 18 to 55 years) who were naïve to antiretroviral therapy and were receiving HIV care at the Partnership Comprehensive Care Practice (Philadelphia, PA). We excluded patients with CD4+ T cell counts less than 350 cells/uL, reasoning that these patients should be encouraged to immediately begin antiretroviral therapy. We also excluded patients with evidence of renal insufficiency, elevated hepatic transaminases, and a body mass index less than 19 kg/m^2^ or greater than 35 kg/m^2^. Written informed consent was obtained from all patients and the study was approved by the institutional review board of Drexel University College of Medicine.

After an overnight fast, 600 mg rifampin was orally administered to each participant. Blood samples were collected prior to ingestion and then at 0.5, 1, 1.5, 2, 2.5, 3, 4, 6, and 8 hours following ingestion. Following centrifugation, serum samples were stored at −80°C and shipped to the Infectious Disease Pharmacokinetics Laboratory at the University of Florida for measurement of rifampin concentrations, using a validated high-performance liquid chromatography assay. On the morning of the pharmacokinetic visit, prior to the administration of rifampin, blood samples were collected for immunologic assays, which were performed in the Kutzler Laboratory.

The human sCD14 Quantikine immunoassay (DC140, R&D Systems, Minneapolis, Minnesota, USA) was performed using commercially available ELISA assay kits according to the manufacturer's protocols on plasma diluted to 50% and 0.5% and undiluted, respectively. The final concentration of sCD14 is based on values enumerated using the standard curve that is run on each ELISA plate. All assays were performed and each test was determined in duplicate, and the average of sCD14 values was calculated. Flow cytometry was performed to measure surface markers of immune activation on CD4+ and CD8+ T lymphocytes, including CD38, HLA-DR, and CD57. The expression of CD38 and HLA-DR on CD8+ T cells is a summary measure of HIV-associated immune activation [15], and CD57 expression on CD8+ T cells is a measure of immune senescence in chronic HIV infection which identifies cells on the pathway of apoptosis [18]. Briefly, for flow cytometry analysis, PBMCs were isolated from whole blood by Ficoll-Hypaque (GE Healthcare) density gradient centrifugation and cryopreserved in freezing media that contained human serum albumin (HSA, Gemini Bio-Products), Hetastarch, and 10% DMSO. At the time of flow cytometry stainings, PBMCs were thawed, washed, and rested for 30 min at 37°C in Benzonase diluted in R10 (RPMI 1640 medium [Sigma Aldrich] containing 10% FBS, 50 IU/ml penicillin and 50 *μ*g/ml streptomycin, and 10 mM HEPES [Life Technologies]). PBMCs were counted using a Countess Automated Cell Counter (Invitrogen) and plated to a concentration of 1 × 106 cells/well in round-bottom 96-well plates. Cells were washed and stained with antibody reagents for 30 min at 20°C. PBMCs were stained with CD3, CD4, CD8a, CD57, CD28, HLA-DR (eBioscience), CD38 (BD), and live/dead staining kit (Invitrogen). Stained cells were washed and resuspended in PBS containing 1% paraformaldehyde. All flow cytometric analyses were conducted within 2 hours after fixation. PBMCs were analyzed on a standardized 18-color LSRFortessa (BD Biosciences), where at least 200,000 total events were collected per run. Antibody capture beads (BD Biosciences) and separate staining with all antibodies used in the panel were run for data compensation. FlowJo 10.0.8r1 (Tree Star) was used for flow cytometric gating analyses. Graphing and statistical analyses were done with Prism 6 (GraphPad Inc.).

Participants returned for a second visit for the performance of intestinal absorption and permeability assays. The D-xylose assay measures the absorptive capacity of the small intestine and is used to determine whether defects in the intestinal epithelium are responsible for malabsorption of drugs or nutrients. After an overnight fast, 25 g of D-xylose was administered in 250 mL of water, and a mixture of lactulose (5 g) and mannitol (1 g) was administered in 250 mL of water. All urine was collected for the subsequent 5 hours. The D-xylose spectrophotometric assay [19] was performed by LabCorp (Burlington, NC), with less than 4.0 g D-xylose collected over 5 hours indicative of impaired absorption. Intestinal permeability was measured with the lactulose/mannitol assay. Lactulose and mannitol are both poorly absorbed, nondigestible carbohydrates, and an elevated ratio indicates either increased intestinal permeability to large molecules between the intestinal epithelial cells (lactulose) or decreased transcellular absorption (mannitol), reflecting a loss of absorptive surface area [20]. The lactulose/mannitol ratio assay was performed on an aliquot of the 5-hour urine collection (Genova Diagnostics, Ashville, NC), based on enzymatic carbohydrate assays [21, 22]. According to the laboratory reference range, a ratio of percent lactulose excretion to percent mannitol excretion greater than 0.11 was supportive of increased intestinal permeability.

The goal of the statistical analysis plan was to describe the variability in measures of immune activation and intestinal function assays and to explore the relationship between immune activation, intestinal function, and rifampin pharmacokinetic parameters. Continuous measures were summarized with median and range, and distributions of immune activation parameters were examined graphically in a scatterplot matrix. Noncompartmental analysis was performed to describe the rifampin peak concentration (*C*_max_), the time to peak concentration (*T*_max_), and the area under the rifampin concentration-versus-time curve during the sampling period (AUC_0–8_) for each participant. Given the limited number of participants, we did not perform compartmental pharmacokinetic modeling or adjusted analyses. All statistical analysis was performed in R version 3.1.1, with noncompartmental analysis performed using the PK package [23].

## 3. Results

We enrolled 6 participants that completed all study procedures (5 men and 1 woman). The median age was 27 years (range: 22–33 years), and all participants reported non-Hispanic and African-American race and ethnicity. All participants were naïve to antiretroviral therapy at the time of the study visits, with a median CD4+ T cell count of 675 (range: 367–1573). There was one participant with an undetectable HIV viral load, consistent with her clinical status as a long-term nonprogressor.

**Figure 1:**
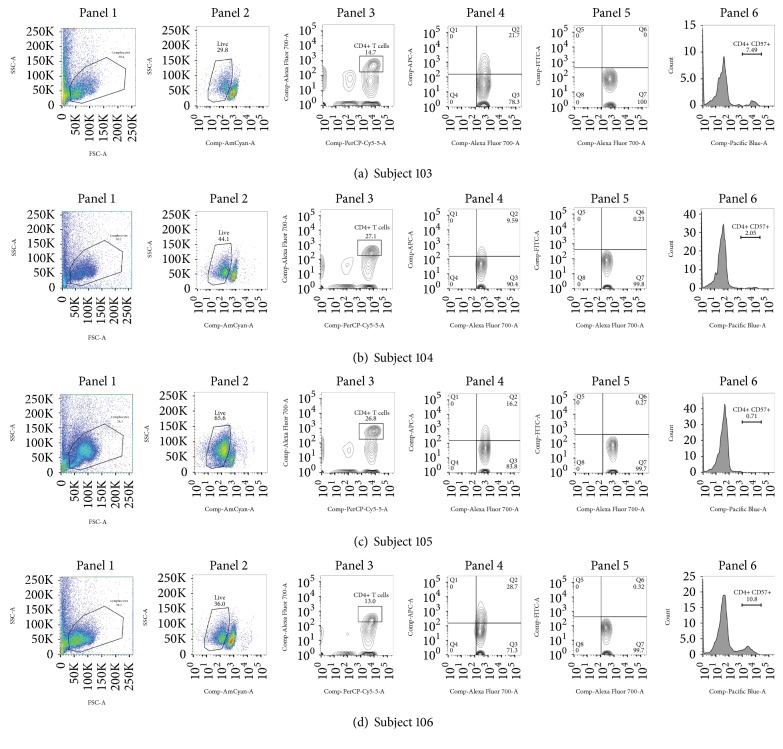
Flow cytometry results for CD4+ T cells. Panel 1: gating of lymphocytes; panel 2: live/dead staining; panel 3: CD3+ CD4+; panel 4: CD4+ HLA-DR+; panel 5: CD4+ CD38+; panel 6: CD4+ CD57+.

**Figure 2:**
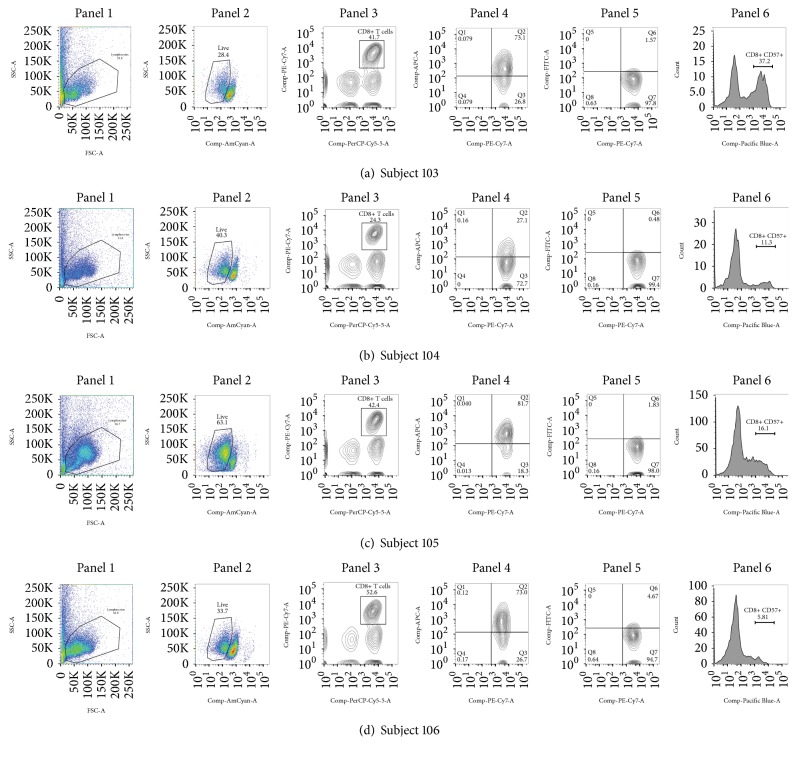
Flow cytometry results for CD8+ T cells. Panel 1: gating of lymphocytes; panel 2: live/dead staining; panel 3: CD3+ CD8+; panel 4: CD8+ HLA-DR+; panel 5: CD8+ CD38+; panel 6: CD8+ CD57+.

The results of the immune activation and microbial translocation assays are shown in Table 1. Flow cytometry was performed in 4 of 6 patients with sufficient peripheral blood mononuclear cells for analysis, as shown in Figure 1 (CD4+ T cells) and Figure 2 (CD8+ T cells). Among all participants, expression of HLA-DR on both CD4+ and CD8+ T cells was greater than expression of CD38. Surprisingly, we did not observe any CD8+ T cells that were dual positive for CD38+ and HLA-DR+. Summaries of the flow cytometry surface markers are included in Table 1.

Based on the threshold of 4.0 g of D-xylose over a 5-hour urine collection period, only one participant (Subject 104) met criteria for impaired intestinal absorption, with a total urinary D-xylose excretion of 3.3 g (normal > 4.0 g). The 5-hour urine lactulose/mannitol ratio was elevated in one participant (Subject 102) with a lactulose/mannitol ratio of 0.11, indicating an increase in intestinal permeability (normal < 0.11).

**Figure 3:**
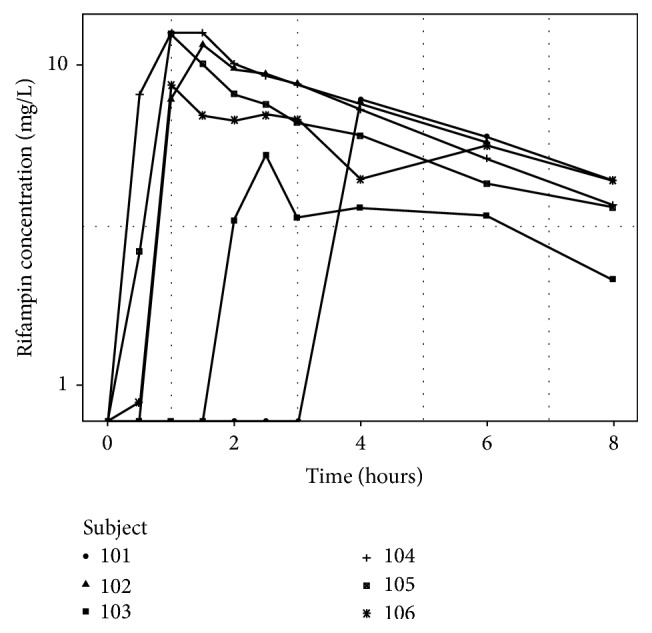
Individual plots of serum rifampin concentration-versus-time curves.

The rifampin serum concentrations versus time are shown in Figure 3. Overall, 4 of 6 participants had a *C*_max_ value greater than the target of 8 mg/L, with a median *C*_max_ of 10.1 mg/L (range: 5.3 to 12.5). The median AUC_0–8_ was 43.6 mg·hr/L (range: 23.5 to 57.4). We observed an absorption delay, defined as *T*_max_ greater than 2 hours, in 2 of 6 patients. The median *C*_max_ and AUC_0–8_ in these 2 patients were 6.5 mg/L and 24.7 mg·hr/L, respectively, compared with 12.0 mg/L and 44.9 mg·hr/L among the 4 patients without delayed absorption. Neither of the participants with delayed rifampin absorption demonstrated low intestinal absorptive capacity by D-xylose testing or increased intestinal permeability by the lactulose/mannitol ratio. In an exploratory analysis of the relationship between markers of immune activation and rifampin absorption, we observed that the sCD14 values were elevated among the 2 participants with delayed absorption (median: 1925 ng/mL), compared with the 4 participants without delayed absorption (median: 1252 ng/mL, *p* = 0.06).

## 4. Discussion

In this pilot study of rifampin pharmacokinetics conducted in 6 HIV-infected (tuberculosis-uninfected) individuals naïve to antiretroviral therapy, we observed a trend towards elevated markers of bacterial translocation among 2 of 6 patients with delayed rifampin absorption, compared to 4 of 6 patients without delayed absorption. One explanation for this finding is that sCD14 provides a blood-based biomarker of the shift of enterocytes from nutritive or absorptive to inflammatory functions, and this potential relationship should be investigated in larger studies of HIV/tuberculosis patients. Interestingly, the patient with the greatest absorption delay was the participant classified as a long-term nonprogressor who maintained an undetectable HIV viral load and high CD4+ T cell count despite a 10-year time interval since HIV infection was diagnosed. Absorption delays with rifampin are one motivation for the recommendation to obtain blood samples 2 hours and 6 hours after dosing when performing therapeutic drug monitoring [1]. We did not observe differences in intestinal absorptive capacity or permeability between participants with and without delayed rifampin absorption.

This pilot study had several important limitations. The limited sample size precluded the conduct of compartmental pharmacokinetic analysis using nonlinear mixed effects models, and therefore we were unable to evaluate the individual measures of immune activation as covariate effects on the coefficient of absorption. Rifampin undergoes autoinduction during the first several weeks of therapy, leading to a fall in drug exposure once steady-state pharmacokinetic conditions are reached, and therefore the observed relationship between immune activation and rifampin exposure may be expected to change during the course of therapy. Furthermore, the exclusion of individuals with a body mass index less than 19 kg/m^2^, as well as individuals with active tuberculosis, may limit the generalizability to populations of HIV/tuberculosis patients with advanced disease and more severe malabsorption, as well as the use of a uniform 600 mg dose rather than weight-based dosing [1]. Strengths of the study include the novel performance of intestinal functional assays simultaneous with detailed immunologic and pharmacokinetic assessments, providing a description of the distribution of these assays which supports the design of future studies and the intensive pharmacokinetic sampling design during the absorptive phase.

In summary, we observed significant variability in measures of immune activation, intestinal damage, and bacterial translocation among HIV-infected individuals naïve to antiretroviral therapy. The potential relationship between markers of bacterial translocation and delayed rifampin absorption merits further evaluation in a prospective study of HIV/tuberculosis patients.

## Figures and Tables

**Table 1:** Immune activation, intestinal assays, and rifampin PK measures.

Characteristic	Study participant ID						Median value
	101	102	103	104	105	106	
*Demographics*							
Age (years)	27	25	27	22	33	29	27
Weight (kg)	90	65	117	62	90	84	87
Sex	F	M	M	M	M	M	—
*Immunologic measures*							
CD4+ T cells (per uL)	1573	367	416	724	626	843	675
sCD14 (ng/mL)	2412	1260	1438	1435	954	1243	1347.5
%CD38+|CD4+	^*∗*^	^*∗*^	0	0.2	0.3	0.3	0.3
%HLA-DR+|CD4+	^*∗*^	^*∗*^	21.7	9.6	16.2	28.7	19.0
%CD57+|CD4+	^*∗*^	^*∗*^	7.5	2.1	0.7	10.8	4.8
%CD38+|CD8+	^*∗*^	^*∗*^	1.6	0.5	1.8	4.7	1.7
%HLA-DR+|CD8+	^*∗*^	^*∗*^	73.1	27.1	81.7	73.0	73.1
%CD57+|CD8+	^*∗*^	^*∗*^	37.2	11.3	16.1	5.8	13.7
*Intestinal assays*							
D-Xylose excretion in urine (g, normal > 4.4 g)	4.6	4.4	4.4	3.3	5.0	5.7	4.5 g
Lactulose/mannitol ratio (normal < 0.11)	0.09	0.11	0.08	0.04	0.05	0.06	0.07
*Rifampin PK measures*							
AUC_0–8_ (mg*∗*hr/L)	28.1	43.1	21.2	57.6	46.6	42.4	42.8
*C*_max_ (mg/L)	7.8	11.6	5.3	12.5	12.5	8.7	10.1
*T*_max_ (hours)	4	1.5	2.5	1.5	1	1	1.5
Clearance (L/hr)	10.3	7.2	15.9	7.6	8.1	7.8	8.0
Volume of distribution (L)	76.9	51.7	113.2	48.0	48.0	69.0	60.4

^*∗*^Insufficient number of PBMCs for flow cytometry.

## Abbreviations

HIV: Human immunodeficiency virus
*C*_max_: Maximum concentration
AUC: Area under the curve
*T*_max_: Time to peak concentration.

## Data Access

The datasets analyzed during the current study are available from the corresponding author upon reasonable request.

## References

[1] Nahid P., Dorman S. E., Alipanah N. (2016). Official American Thoracic Society/centers for disease control and prevention/infectious diseases society of America clinical practice guidelines: treatment of drug-susceptible tuberculosis. *Clinical Infectious Diseases*.

[2] Pasipanodya J., Gumbo T. (2011). An oracle: antituberculosis pharmacokinetics-pharmacodynamics, clinical correlation, and clinical trial simulations to predict the future. *Antimicrobial Agents and Chemotherapy*.

[3] Pasipanodya J. G., McIlleron H., Burger A., Wash P. A., Smith P., Gumbo T. (2013). Serum drug concentrations predictive of pulmonary tuberculosis outcomes. *The Journal of Infectious Diseases*.

[4] Tappero J. W., Bradford W. Z., Agerton T. B. (2005). Serum concentrations of antimycobacterial drugs in patients with pulmonary tuberculosis in Botswana. *Clinical Infectious Diseases*.

[5] Dorman S. E., Savic R. M., Goldberg S. (2015). Daily rifapentine for treatment of pulmonary tuberculosis. A randomized, dose-ranging trial. *American Journal of Respiratory and Critical Care Medicine*.

[6] Pasipanodya J. G., Srivastava S., Gumbo T. (2012). Meta-analysis of clinical studies supports the pharmacokinetic variability hypothesis for acquired drug resistance and failure of antituberculosis therapy. *Clinical Infectious Diseases*.

[7] Chigutsa E., Pasipanodya J. G., Visser M. E. (2015). Impact of nonlinear interactions of pharmacokinetics and mics on sputum bacillary kill rates as a marker of sterilizing effect in tuberculosis. *Antimicrobial Agents and Chemotherapy*.

[8] Sahai J., Gallicano K., Swick L. (1997). Reduced plasma concentrations of antituberculosis drugs in patients with HIV infection. *Annals of Internal Medicine*.

[9] Perlman D. C., Segal Y., Rosenkranz S. (2005). The clinical pharmacokinetics of rifampin and ethambutol in HIV-infected persons with tuberculosis. *Clinical Infectious Diseases*.

[10] Façanha M. C., Gondim A. M. B., Pinheiro V. G. F. (2009). Intestinal barrier function and serum concentrations of rifampin, isoniazid and pyrazinamide in patients with pulmonary tuberculosis. *The Brazilian Journal of Infectious Diseases*.

[11] Barroso E. C., Pinheiro V. G., Façanha M. C., Carvalho M. R., Moura M. E., Campelo C. L. (2009). Serum concentrations of rifampin, isoniazid, and intestinal absorption, permeability in patients with multidrug resistant tuberculosis. *The American Journal of Tropical Medicine and Hygiene*.

[12] Pinheiro V. G. F., Ramos L. M. A., Monteiro H. S. A. (2006). Intestinal permeability and malabsorption of rifampin and isoniazid in active pulmonary tuberculosis. *The Brazilian Journal of Infectious Diseases*.

[13] Mehandru S., Tenner-Racz K., Racz P., Markowitz M. (2005). The gastrointestinal tract is critical to the pathogenesis of acute HIV-1 infection. *The Journal of Allergy and Clinical Immunology*.

[14] Brenchley J. M., Price D. A., Douek D. C. (2006). HIV disease: Fallout from a mucosal catastrophe?. *Nature Immunology*.

[15] Liu Z., Cumberland W. G., Hultin L. E., Prince H. E., Detels R., Giorgi J. V. (1997). Elevated CD38 antigen expression on CD8+ T cells is a stronger marker for the risk of chronic HIV disease progression to AIDS and death in the multicenter AIDS cohort study than CD4+ cell count, soluble immune activation markers, or combinations of HLA-DR and CD38 expression. *Journal of Acquired Immune Deficiency Syndromes*.

[16] Sandler N. G., Wand H., Roque A. (2011). Plasma levels of soluble CD14 independently predict mortality in HIV infection. *The Journal of Infectious Diseases*.

[17] Medellín-Garibay S. E., Cortez-Espinosa N., Milán-Segovia R. C. (2015). Clinical pharmacokinetics of rifampin in patients with tuberculosis and type 2 diabetes mellitus: Association with biochemical and immunological parameters. *Antimicrobial Agents and Chemotherapy*.

[18] Brenchley J. M., Karandikar N. J., Betts M. R. (2003). Expression of CD57 defines replicative senescence and antigen-induced apoptotic death of CD8^+^ T cells. *Blood*.

[19] Craig R. M., Atkinson A. J. (1988). d-Xylose testing: A review. *Gastroenterology*.

[20] Ménard S., Cerf-Bensussan N., Heyman M. (2010). Multiple facets of intestinal permeability and epithelial handling of dietary antigens. *Mucosal Immunology*.

[21] Northrop C. A., Lunn P. G., Behrens R. H. (1990). Automated enzymatic assays for the determination of intestinal permeability probes in urine. 1. Lactulose and lactose. *Clinica Chimica Acta*.

[22] Lunn P. G., Northrop C. A., Northrop A. J. (1989). Automated enzymatic assays for the determination of intestinal permeability probes in urine. 2. Mannitol. *Clinica Chimica Acta*.

[23] Jaki T., Wolfsegger M. J. (2011). Estimation of pharmacokinetic parameters with the R package PK. *Pharmaceutical Statistics*.

